# LenVarDB: database of length-variant protein domains

**DOI:** 10.1093/nar/gkt1014

**Published:** 2013-11-03

**Authors:** Eshita Mutt, Oommen K. Mathew, Ramanathan Sowdhamini

**Affiliations:** ^1^International Institute of Information Technology-Hyderabad, Gachibowli, Hyderabad 500032, Andhra Pradesh, India, ^2^National Centre for Biological Sciences (TIFR), UAS-GKVK Campus, Bellary Road, Bangalore 560065, Karnataka, India and ^3^SASTRA University, Tirumalaisamudram, Thanjavur 613401, Tamil Nadu, India

## Abstract

Protein domains are functionally and structurally independent modules, which add to the functional variety of proteins. This array of functional diversity has been enabled by evolutionary changes, such as amino acid substitutions or insertions or deletions, occurring in these protein domains. Length variations (indels) can introduce changes at structural, functional and interaction levels. LenVarDB (freely available at http://caps.ncbs.res.in/lenvardb/) traces these length variations, starting from structure-based sequence alignments in our Protein Alignments organized as Structural Superfamilies (PASS2) database, across 731 structural classification of proteins (SCOP)-based protein domain superfamilies connected to 2 730 625 sequence homologues. Alignment of sequence homologues corresponding to a structural domain is available, starting from a structure-based sequence alignment of the superfamily. Orientation of the length-variant (indel) regions in protein domains can be visualized by mapping them on the structure and on the alignment. Knowledge about location of length variations within protein domains and their visual representation will be useful in predicting changes within structurally or functionally relevant sites, which may ultimately regulate protein function. Non-technical summary: Evolutionary changes bring about natural changes to proteins that may be found in many organisms. Such changes could be reflected as amino acid substitutions or insertions–deletions (indels) in protein sequences. LenVarDB is a database that provides an early overview of observed length variations that were set among 731 protein families and after examining >2 million sequences. Indels are followed up to observe if they are close to the active site such that they can affect the activity of proteins. Inclusion of such information can aid the design of bioengineering experiments.

## INTRODUCTION

Protein domains are functional and compact structural units of protein, which evolve with time by incorporating various changes in the form of amino acid substitutions and insertions. Insertion/deletion of contiguous stretch of amino acids (indels) can induce structural modifications, which can eventually impart functional diversity or affect stability or differences in quaternary arrangements of these protein domains. Analysis of indels among sequence homologues of domain superfamilies will provide an early understanding of the evolutionary changes of protein function from a handful of protein folds (1).

Occurrence of indels is a continuous process and found frequently in loop regions, especially as ‘nested forms’ into previously inserted regions (2) or observed as substructural regions in the minimum core of protein domain scaffold, termed as ‘structural embellishments’ (3). Analysis of such embellishments in HIGH-signature proteins, UspA, and PP-ATPase (HUP) domain superfamily showed that indels are usually located sequentially far apart, but are spatially proximate and form subdomains, which further fine-tune the diverse functions of different members of the same superfamily (4). Recent studies by our group and others had focussed on the role played by indels in affecting structure, function (5,6) and oligomeric status (7) of a protein, while emphasizing that fixation of such indels in genome is highly context-dependent (8). Introduction of short indels within active-site loops caused emergence of other enzymatic capabilities (9,10) or led to conformational switches, as in the case of C2A domain in Piccolo protein (11). Although most of the studies have been carried out on full-length proteins, it will be interesting to trace these length variations (indels) within protein domain boundaries, given the fact that protein domains are functionally independent modules (12).

LenVarDB is a database of length-variant protein domains that documents length variation statistics within homologues of each SCOP (Structural Classification of Proteins) (13) entry, considered within our PASS2 (Protein Alignments organized as Structural Superfamilies) database (14). Alignments of structural and sequence domains are annotated for their length-variant regions (indel) or ‘structurally conserved blocks’ (SSB) (15) in LenVarDB. Starting from 731 multi-membered PASS2, consisting of 8394 individual protein domains, non-redundant sequence database (NCBI-NR) was queried to obtain 2 730 625 sequence homologues, whose analysis in turn led to the detection of 192 742 indels (Supplementary Table S1). Unlike other indel databases (such as IndelFR (16), IndelPDB (17), IndelScan (18), which deal only with pairwise alignment to detect indels), we introduce sequences in a structure-based multiple sequence alignment, thus extracting evolutionary insight from the sequence space in detecting indel-prone regions. LenVarDB is a useful compendium to gain insight about the effects of length variations (indels) on the structure and function of a protein domain.

## MATERIALS AND METHODS

### Inclusion of sequence homologues and their alignment

In all, 731 superfamilies (with >2 members) from PASS2 version 4 (14) were used as an initial dataset, whose sequences (members of each superfamily) were queried against ‘NCBI NR (June 2012)’ database by using an in-house standardized pipeline explained elsewhere (19). This pipeline had stage-specific filters and had been optimized to encourage the collection of length-variant homologues without encountering false positives. Superfamilies were classified into groups (as length-deviant, length-rigid or length-normal) based on their extent of length variation (details in Supplementary Methods). Non-redundant homologues for each superfamily member were included in an alignment, which were importantly guided by structure-based sequence alignments obtained from PASS2, as it was more accurate than using any sequence-based alignment (20) and to reduce the random insertion of gaps within secondary structures. Length-variant (indel) regions, determined from these alignments, along with SSB obtained from conserved units of structure in proteins (CUSP) algorithm (15), were mapped on the protein domain structures (details in Supplementary Methods). Statistical tests were carried out using *R* package (21).

### Database design

Web interface of LenVarDB was made interactive and efficient by using HTML, CSS, JavaScript, AJAX and JQuery. The backend services were developed using PERL, PERL-CGI and MySQL in Apache2 environment, and data analysis was performed by BioPERL modules (22) and atm2seq (23) package. Data visualization was aided by PERL-GD::Graphs module, and representation of structure and alignments was implemented using Jmol (Jmol: an open-source Java viewer for chemical structures in 3D, http://www.jmol.org/) and Jalview (24).

## RESULTS AND DISCUSSION

In this study, we have traced the length variations within a protein domain using SCOP (13)-derived PASS2 and their structure-based alignments. To obtain comprehensive pointers about the role and position of length variations across superfamilies, we have introduced sequence homologues apart from their structural entries, in the respective superfamilies. LenVarDB database has been organized in a user-friendly manner, and various features that have been implemented will be helpful to a user in determining the length variation status of their query protein.

### Organization of the data (browse option)

Superfamilies, along with their respective members, are organized in the following two ways: first, according to groups of length variation, and second, in accordance to SCOP classes.

### Information about each superfamily

**Figure 1.**
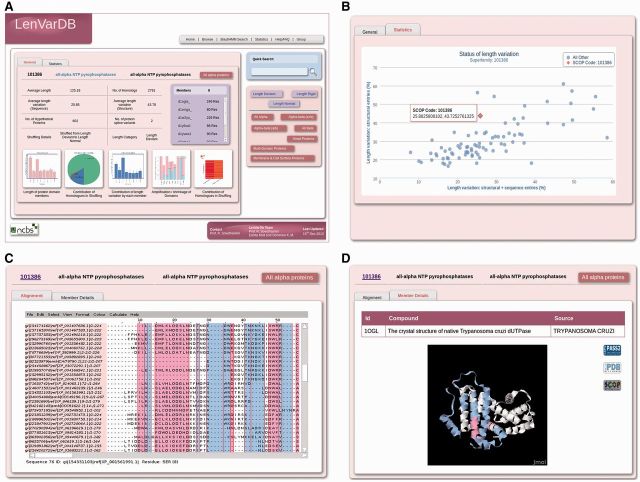
Illustration of the features of the database. (**A**) Length variation details at superfamily level, which have been comprehensively described by the graphs. (**B**) Correlation plot for finding length-variant status of a particular superfamily in the length-deviant group (as shown here). (**C**) Multiple sequence alignment annotated by length-variant regions (indels in blue) and SSB (in pink). (**D**) Details of superfamily member and the length-variant regions and SSBs marked over the member structure to aid visualization in spatial format. Cross-references to PDB, PASS2 and SCOP for each entry are provided.

The superfamily page summarizes the length variation features computed for each superfamily and its members at the time of database creation. Length variation is calculated at two levels, one with only the structural members of superfamily [average length variation (structure)] and the other with all the sequence homologues collected [average length variation (sequence)]. The length-variant group to which the particular superfamily belongs to (according to structural entries) is noted, and information about any shuffling event (within length-variant groups) is mentioned. A separate panel enlists each member and its length, which is also hyperlinked to ‘Member page’. Dynamically created graphs are provided for easy visualization of the data provided. Length variation status of each homologue and its contribution in maintaining or changing the length variation status of the superfamily is featured in an interactive scatter plot. The relation between length variation and sequence identity (%) of every homologue collected for every superfamily member is represented by a heat map. Fluctuations in length, captured in the form of amplification/shrinkage from the domain length, are shown for all members in the form of a stacked bar graph. An estimate of hypothetical proteins and splice variants (found in the homologue sets) is also provided (Figure 1). To view the distribution of homologues from a taxonomy perspective, a ‘Taxonomy tab’ has been created to classify the homologues as per their TaxID (NCBI) and annotate average and range of length variation accumulated for the major domains of life, namely, archaea, bacteria and eukaryotes (Supplementary Data).

### Descriptions of every superfamily member

Length variations, recorded across the homologues of a certain member in the form of indels (please see Supplementary Methods), are mapped on the multiple sequence alignment and 3D structure. SSBs (from CUSP algorithm) are also marked in a similar manner (Figure 1B). Users can interactively visualize the length-variant regions on the 3D structure of a protein domain. Each superfamily member is also cross-linked to widely known databases like Protein Data Bank (25), PASS2 and SCOP for detailed structural and domain level information.

### Searching and aligning query of interest to superfamily

Keyword search option has been integrated in the home page for easy access to any superfamily or its member. An amino acid sequence of a query protein can be used to search for the nearest superfamily by the integrated PSI-BLAST and HMMER programs. Further, the query can be aligned with the pre-aligned set of homologues corresponding to superfamily members.

### Accessing information as downloads

Length-variance information about each superfamily member and their homologues is available for download. For each member, multiple sequence alignment with their homologues, Genbank identifier list of hypothetical proteins and listing of length variation (%) and sequence identity (%) of every homologue with respect to the superfamily member can be accessed.

### Assistance for users

A user-friendly help page has been created to guide the access and features of the database. A video tour of the database has also been embedded in the home page for the same purpose [Supplementary Movie S1 (http://caps.ncbs.res.in/download/lenvardb/)]. Frequently asked questions enlisted will enable users in analysing their query proteins from a length variation perspective.

### Statistical analysis of LenVarDB

Length variation parameter has been used for statistical analyses (as shown in Supplementary Data) and integrated as ‘Statistics’ page in the database.

Presence of insert domain has been analysed and found to occupy only 2% of the PASS2 starting dataset (8970 superfamily members), which gives the user an insight about one of the causes of length variations in LenVarDB (Details in Supplementary Table S2 and Supplementary Figure S6). The range of length variation [shown as box plots to represent the range of length variation (%)] in eukaryotic homologues is slightly higher than bacterial and archaeal homologues (Supplementary Figure S8).

## APPLICATION OF THE DATABASE

Detection of multiple length variations (indels) in their spatial orientation within a protein domain structure can be useful in focusing on the deviations caused from its usual structure and function. Study of length variations in a homologue (*Geobacillus S. Y412MC61* dUTPase [gi|261418089] from all-alpha NTP pyrophosphatase SCOP superfamily) of *Campylobacter jejuni* dUTPase reveals the presence of a 20 residue-insert near the substrate binding domain and dimerization interface, which can regulate its functionality or oligomerization status (Supplementary Data). Further biochemical characterization can show if the presence of these indels (in the homologue) interferes with the pathogenic nature of this dUTPase in *C. jejuni*, which is known to be a gastric pathogen.

**Figure 2.**
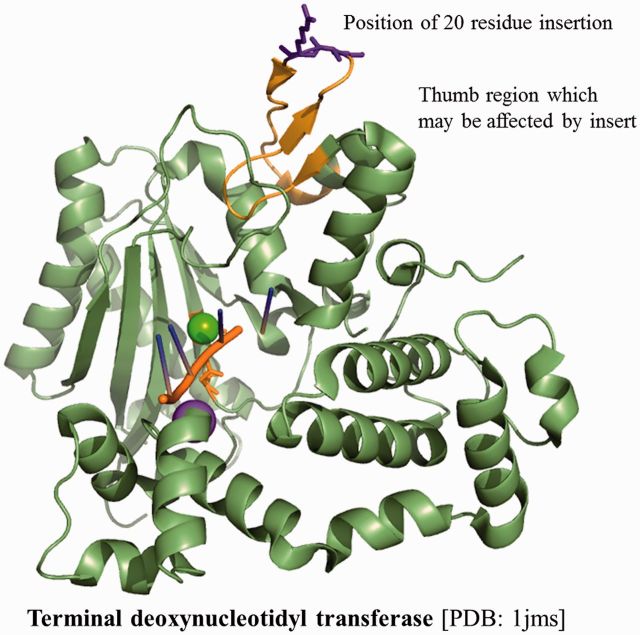
Position of insertion and deletion traced on mouse terminal deoxynucleotidyl transferase homologue can aid in structural and modelling studies.

Another use of LenVarDB can be in tracking length-variant regions in newly sequenced proteins. An example of aligning and structure-mapping of an isoform of terminal deoxynucleotidyl transferase (gi|112734847 from deoxynucleotidyl transferase superfamily) is illustrated (Figure 2). Literature suggests that the indel found near the carboxy-terminal end of this domain significantly alters its function (26).

LenVarDB is a comprehensive resource designed for researchers interested in protein domain biology. This database systematically and automatically gathers sequences, aligns them to pre-existing structure-guided PASS2 alignments and derives indel (length-variant)-prone regions of protein domain superfamilies. The web interface has been made interactive and readily available for the scientific community. The key advantage of LenVarDB over other indel databases is that it introduces evolutionary insights from all the closely and distantly related sequence homologues. LenVarDB allows users to identify the indel-prone regions, which can enable rational design of bioengineering experiments.

## SUPPLEMENTARY DATA

Supplementary Data are available at NAR Online, including [27–32].
